# Reciprocal Fluctuations in Lipoprotein Lipase, Glycosylphosphatidylinositol-Anchored High-Density Lipoprotein-Binding Protein 1, and Hepatic Triglyceride Lipase Levels in the Peripheral Bloodstream Are Correlated with Insulin Resistance

**DOI:** 10.3390/nu17111880

**Published:** 2025-05-30

**Authors:** Takumi Nagasawa, Koji Sakamaki, Akihiro Yoshida, Hiroki Machida, Fumitaka Murakami, Mari Hashimoto, Takahito Shinohara, Masami Murakami, Katsuhiko Tsunekawa, Takao Kimura

**Affiliations:** 1 Department of Clinical Laboratory Medicine, Gunma University Graduate School of Medicine, Showa-Machi 3-39-22, Maebashi 371-8511, Gunma, Japan; t_nagasawa@gunma-u.ac.jp (T.N.); kenshin2521@hidaka-kai.com (K.S.); ayossie10@gunma-u.ac.jp (A.Y.); h.machida@gunma-u.ac.jp (H.M.); takahito.shinohara.49@gmail.com (T.S.); mmurakam@gunma-u.ac.jp (M.M.); ktsune@gunma-u.ac.jp (K.T.); 2 Clinical Laboratory Center, Gunma University Hospital, Showa-Machi 3-39-22, Maebashi 371-8511, Gunma, Japan; f.murakami.1202@gunma-u.ac.jp (F.M.); mari.ha@gunma-u.ac.jp (M.H.); 3 Hidaka Hospital, Nakao-Machi-886, Takasaki 370-0001, Gunma, Japan; 4 Medical Education and Development, Gunma University Graduate School of Medicine, Showa-Machi 3-39-22, Maebashi 371-8511, Gunma, Japan; 5 Graduate School of Food and Population Health Science, Gunma University, Aramaki-Machi 4-2, Maebashi 371-8510, Gunma, Japan; 6 General Health Support Center, Gunma University, Aramaki-Machi 4-2, Maebashi 371-8510, Gunma, Japan

**Keywords:** BMI, waist circumference, exercise habit, insulin resistance, LPL, GPIHBP1, HTGL

## Abstract

**Background/Objectives:** This study aimed to identify the regulatory system of lipoprotein lipase (LPL), glycosylphosphatidylinositol-anchored high-density lipoprotein (HDL)-binding protein 1 (GPIHBP1), and hepatic triglyceride lipase (HTGL) in the peripheral bloodstream. **Methods**: In total, 207 individuals (100 males and 107 females) who were diagnosed with normal glucose tolerance or prediabetes during their comprehensive health checkup were investigated. **Results**: Circulating LPL levels were positively correlated with the GPIHBP1 and HDL-cholesterol (HDL-C) levels, and negatively correlated with body mass index (BMI), waist circumference (WC), Homeostasis Model Assessment of Insulin Resistance (HOMA-IR) scores, triglyceride–glucose index, and triglyceride, fasting insulin, ferritin, and C-reactive protein (CRP) levels. The GPIHBP1 level was positively correlated with LPL and HTGL levels, and negatively correlated with estimated glomerular filtration rate (eGFR). The HTGL level was positively correlated with BMI, WC, HOMA-IR score, and GPIHBP1, low-density lipoprotein cholesterol (LDL-C), fasting insulin, and ferritin levels. Meanwhile, it was negatively correlated with HDL-C levels. The multiple regression analysis revealed that the circulating LPL level was independently affected by BMI, red blood cell (RBC) count, GPIHBP1, fasting plasma glucose (FPG), fasting insulin, HDL-C, CRP, and ferritin levels. The GPIHBP1 level was independently affected by age, eGFR, FPG, LPL, and HTGL levels and RBC count. The HTGL level was independently affected by WC, GPIHBP1 and LDL-C levels. **Conclusions**: LPL and HTGL levels reflect insulin resistance. In particular, individuals with a greater insulin resistance present with a lower LPL level and a higher HTGL level. An increased GPIHBP1 level might compensate for decreased LPL levels due to insulin resistance.

## 1. Introduction

Lipoprotein lipase (LPL) is a key player in the intravascular hydrolysis of triglyceride -rich lipoproteins, such as chylomicrons and very-low-density lipoprotein (VLDL) cholesterol [1,2,3]. LPL decreases triglyceride (TG) levels and increases high-density lipoprotein cholesterol (HDL-C) levels in the peripheral bloodstream [1,2,3]. Previously, clinical research on the correlation between LPL levels and disease pathophysiology was performed by measuring LPL activity in the blood samples taken after the intravenous administration of heparin. However, studies using blood samples collected after heparin administration are non-physiological. Our studies and others have reported that the significance of measuring circulating LPL concentrations without heparin administration using a highly sensitive enzyme-linked immunosorbent assay (ELISA) is similar to that of measuring LPL activity after heparin administration [4,5,6,7,8,9,10,11,12,13,14,15]. These reports have shown that circulating LPL concentrations without heparin administration are related to metabolic diseases, such as dyslipidemia, diabetes, hypertension, cardiovascular disease (CVD), and fatty liver [4,5,6,7,8,9,10,11,12,13,14,15]. However, the regulatory mechanism of circulating LPL levels in the peripheral bloodstream remains unclear.

LPL is synthesized in the skeletal muscle, cardiac muscle, adipose tissue, and macrophages [3]. LPL synthesized in these peripheral tissues is anchored to the peripheral vascular lumen by glycosylphosphatidylinositol-anchored high-density lipoprotein-binding protein 1 (GPIHBP1) via transfer by heparan-sulfate proteoglycan [16,17,18,19,20]. An ELISA was developed to measure GPIHBP1 levels to analyze the regulatory mechanism of LPL in the blood vessels [21]. Results showed a positive correlation between the circulating LPL and GPIHBP1 levels [22]. However, the regulatory mechanism of circulating GPIHBP1 levels in the peripheral bloodstream remains unclear.

Hepatic triglyceride lipase (HTGL) hydrolyzes TG and phospholipids in the chylomicron, VLDL, and HDL. HTGL is synthesized in hepatocytes and is present at the surface of the liver sinusoidal capillaries, bound to heparan-sulfate proteoglycan [23,24]. The regulatory mechanism of HTGL, similar to that of LPL, has been analyzed by measuring the HTGL activity in the blood after heparin administration [23,24]. A highly sensitive ELISA was established for measuring HTGL levels. Further, the regulatory mechanisms of HTGL concentrations in the peripheral bloodstream under physiological conditions without heparin administration are currently being investigated [25]. Both HTGL and LPL hydrolyze TG. However, they have different characteristics. A previous study has reported that high LPL levels are correlated with a low risk of coronary artery disease. Meanwhile, high HTGL levels are correlated with a high risk of coronary artery disease [15]. Nevertheless, the regulatory mechanism of circulating HTGL levels in the peripheral bloodstream remains unclear. Further, the reciprocal regulatory mechanism between LPL/GPIHBP1 and HTGL levels in the bloodstream remains to be elucidated. These findings suggest a correlation between the pathophysiological machinery of metabolic disease, such as overweight, obesity, diabetes, dyslipidemia, and CVD, and LPL/GPIHBP1 and HTGL levels. Overweight, obesity, diabetes, dyslipidemia, and CVD are closely related to insulin resistance [26]. Notably, the capacity for insulin secretion declines with age in Japanese individuals. Thus, attention should be paid to the assessment of insulin resistance using the Homeostasis Model Assessment of Insulin Resistance (HOMA-IR) [27]. The triglyceride–glucose (TyG) index, which has attracted attention in recent years as an index of insulin resistance that does not use insulin as a variable, was also evaluated [28,29]. This study aimed to validate the regulatory mechanisms of circulating levels of LPL, GPIHBP1, and HTGL in the peripheral bloodstream. To identify such mechanisms, we analyzed LPL, GPIHBP1, HTGL and insulin resistance in middle-aged Japanese individuals.

## 2. Materials and Methods

### 2.1. Study Population

In total, 231 Japanese individuals (116 men and 115 women) who underwent a comprehensive medical examination at Hidaka Hospital in Takasaki, Japan, in 2016 voluntarily participated in this study. Written informed consent was obtained from all participants before participation.

Data on smoking history, exercise habits, and medication use of all participants during the comprehensive medical examination were evaluated. In this study, of 231 participants, 8 who were diagnosed with or undergoing treatment for type II diabetes (T2DM) were excluded. In addition, we excluded 16 subjects taking fibrates. Thus, 207 participants (100 males and 107 females) were included in the final analysis. This study was approved by the ethics committee of Hidaka Hospital (approval no. 127, approval date: 5 April 2016), and was performed in accordance with the ethical principles of the Declaration of Helsinki. This study was also approved by the Gunma University Ethical Review Board for Medical Research Involving Human Subjects (approval no. 2017-219, approval date: 2 April 2018). Further, it adhered to the Declaration of Helsinki regarding all ethical and confidentiality considerations.

### 2.2. Anthropometric Measurement and Laboratory Analyses

The height and weight of the participants were measured to calculate the BMI. BMI was calculated as weight divided by height in meters squared (kg/m^2^). Waist circumference (WC) was measured to the nearest 0.5 cm using a tape measure placed horizontally at the level of the umbilicus while the participants exhaled quietly [30]. Systolic blood pressure (SBP) and diastolic blood pressure (DBP) were also measured, and blood samples were collected from the antecubital vein while the participant was sitting after an overnight fast. The serum total cholesterol (TC), HDL-C, TG, and low-density lipoprotein cholesterol (LDL-C) concentrations were measured using enzymatic assays with an automatic analyzer (TBA-c8000; Canon Medical Systems Corporation, Otawara, Japan). Fasting plasma glucose (FPG) concentrations were assessed using the glucose oxidase method, and hemoglobin A1c (HbA1c) levels were examined with high-performance liquid chromatography using automatic analyzers (GA 08 II; A&T and HLC-723G9; Tosoh Corporation, Tokyo, Japan). Red blood cell (RBC) count, hemoglobin and hematocrit levels, white blood cell (WBC) count, and platelet count were measured using an automatic analyzer (XT-1800i; Sysmex Corporation, Kobe, Japan). The serum C-reactive protein (CRP) and ferritin concentrations were investigated using a latex-enhanced turbidimetric immunoassay with the same analyzer. Serum insulin concentrations were measured using chemiluminescence immunoassay (AIA-2000 LA; Tosoh, Tokyo, Japan) [31,32]. The serum LPL concentrations were measured using a sandwich ELISA (SEKISUI MEDICAL Co., Ltd., Tokyo, Japan) [32]. The GPIHBP1 and HTGL levels were measured using the ELISA (IBL Co., Ltd., Fujioka, Gunma, Japan).

### 2.3. Statistical Analysis

Data were expressed as the median with the 25–75th percentile. The Kruskal–Wallis test and the Dunn’s test with Bonferroni adjustment were performed to compare two groups divided by exercise habit. The HOMA-IR and Homeostasis Model Assessment of β-Cell Function (HOMA-β) scores [33], TyG index [28,29], and estimated glomerular filtration rate (eGFR) [34] were calculated. Significant between-groups differences were identified using the Mann–Whitney U test. Each parameter was compared using Bonferroni’s method, chi-squared test, and one-way analysis of variance followed by Tukey’s post hoc tests. Spearman’s correlation analyses were checked separately for males, females, normal glucose tolerance (NGT), and impaired fasting glucose (IFG). A multiple regression analysis using the stepwise forward selection method was conducted on each variable to examine the relative contribution to circulating LPL, GPIHBP1, and HTGL levels. *p*-values of <0.05 indicated statistically significant differences and correlations. All statistical data were analyzed using StatFlex version 7 (Artech, Osaka, Japan).

## 3. Results

### 3.1. Characteristics of the Participants

According to the Japanese guidelines (Supplementary Figure S1), the 207 participants presented with NGT, high-normal FPG, IFG, impaired glucose tolerance (IGT), and combined glucose intolerance [35]. IGT and combined glucose intolerance could not be identified because the assessment requires patients to undergo the oral glucose tolerance test (Supplementary Figure S1). Of the 207 participants in this study, 153 (73.9%) were classified as NGT and 54 (26.1%) classified as IFG. Table 1 shows the characteristics of the participants. The median ages of all the participants, male participants, and female participants were 58 (range: 49–64), 57 (range: 50–64), and 58 (range: 49–64) years, respectively. The median HbA1c level was 5.8% (range: 5.5–6.1%) (Table 1). All participants presented with either NGT or prediabetes, but not with diabetes, based on the HbA1c and FPG levels according to the Japanese guidelines and the American Diabetes Association criteria [12,13,16]. Compared with the female participants, the male participants had a higher BMI, WC, TyG index, RBC and WBC count, DBP, GPIHBP1, TG, FPG, ferritin, and CRP level. Further, the male participants had lower LPL, TC, and HDL-C levels and eGFR than the female participants (Table 1). The participants taking statins had significantly higher BMI, WC, HbA1c, and TC levels than those not taking statins (Supplementary Table S1). The participants taking anti-hypertensive drugs had significantly higher GPIHBP1 levels than those not taking anti-hypertensive drugs (Supplementary Table S1).

### 3.2. Correlation Between Circulating Levels of LPL, GPIHBP1, and HTGL and Clinical and Laboratory Parameters

Table 2 shows the correlation between circulating levels of LPL, GPIHBP1, and HTGL and each parameter in 207 study participants. The circulating LPL level was positively correlated with GPIHBP1 and HDL-C levels, and was negatively correlated with BMI, WC, HOMA-IR score, TyG index, RBC and WBC count, TG, FPG, fasting insulin, ferritin, and CRP levels. GPIHBP1 was positively correlated with LPL and HTGL levels, and was negatively correlated with eGFR. HTGL was positively correlated with HOMA-IR score, BMI, WC, and GPIHBP1, LDL-C, fasting insulin, and ferritin levels, and was negatively correlated with HDL-C levels. These results indicate that individuals with a higher insulin resistance have a lower LPL level and a higher HTGL level.

Among 100 male participants, the circulating LPL level was positively correlated with GPIHBP1 and HDL-C levels, and was negatively correlated with BMI, WC, TyG index, TG, ferritin and CRP levels (Table 2). GPIHBP1 was positively correlated with LPL and HTGL levels, and was negatively correlated with eGFR (Table 2). HTGL was positively correlated with GPIHBP1, TC, and LDL-C levels, and was negatively correlated with eGFR (Table 2).

Among 107 female participants, the LPL level was positively correlated with GPIHBP1 and HDL-C levels, and was negatively correlated with BMI, fasting insulin, FPG, HOMA-β and HOMA-IR score, ferritin, CRP and WBC count (Table 2). GPIHBP1 was positively correlated with age, LPL and LDL-C levels, and was negatively correlated with eGFR (Table 2). HTGL was positively correlated with BMI, WC, LDL-C, fasting insulin, ferritin, CRP, HOMA-β and HOMA-IR score, and was negatively correlated with HDL-C levels (Table 2).

### 3.3. Correlation Between the TyG Index and Clinical and Laboratory Parameters

Among 207 study participants, the TyG index was positively correlated with age, HOMA-IR score, RBC and WBC count, BMI, WC, TG, LDL-C, fasting insulin, FPG, HbA1c, ferritin, and CRP levels. It was negatively correlated with LPL and HDL-C levels and eGFR (Table 2).

Among 100 male participants, TyG index was positively correlated with BMI, WC, TG, fasting insulin, FPG, HOMA-IR, WBC count, ferritin, and CRP levels, and was negatively correlated with LPL and HDL-C levels (Table 2).

Among 107 female participants, TyG index was positively correlated with age, BMI, WC, TC, TG, LDL-C, FPG, HbA1c, HOMA-IR, and WBC count, and was negatively correlated with HDL-C levels (Table 2).

### 3.4. Comparison of LPL, GPIHBP1, and HTGL and Clinical and Laboratory Parameters Between Normal Glucose Tolerance and Impaired Fasting Glucose Groups

Among 207 participants, BMI, WC, LDL-C, FPG, HbA1c, TyG index, and WBC count were significantly higher in the IFG group than in the NGT group, while LPL, HDL-C, and HOMA-β score were significantly lower (Table 3). Of the 100 male participants, 70 (70%) were classified as NGT and 30 (30%) were classified as IFG (Table 3). TC, LDL-C, FPG, and HbA1c were significantly higher in the IFG group than in the NGT group (Table 3). Of the 107 female participants, 83 (77.6%) were classified as NGT and 24 (22.4%) were classified as IFG (Table 3). Age, BMI, WC, SBP, TG, LDL-C, FPG, HbA1c, and TyG index were significantly higher in the IFG group than in the NGT group, while LPL, HDL-C, and HOMA-β score were significantly lower (Table 3).

Compared with the female group, the male group in the NGT group had significantly higher BMI, WC, SBP, DBP, TG, FPG, TyG index, ferritin, CRP, RBC and WBC count, and significantly lower LPL, TC, HDL-C, and eGFR (Table 3).

The male group with IFG had a significantly higher RBC count and was significantly younger than the female group (Table 3). These findings suggest that circulating LPL and HTGL levels are more affected by insulin resistance in females than in males.

Among 153 participants classified as NGT, the circulating LPL level was positively correlated with GPIHBP1, TC, and HDL-C levels, and was negatively correlated with BMI, WC, TG, fasting insulin, FPG, HOMA-β and HOMA-IR score, TyG index, ferritin, RBC and WBC count, and CRP levels (Table 4). GPIHBP1 was positively correlated with age and LPL levels, and was negatively correlated with eGFR (Table 4). HTGL was positively correlated with BMI, WC, LDL-C and CRP levels, and was negatively correlated with HDL-C levels (Table 4). TyG index was positively correlated with age, BMI, WC, TG, LDL-C, fasting insulin, FPG, HOMA-IR score, ferritin, RBC and WBC count, while it was negatively corelated with LPL and HDL-C (Table 4).

Among 54 participants classified as IFG, the circulating LPL level was negatively correlated with TG, TyG index, and ferritin levels (Table 4). GPIHBP1 was negatively correlated with HDL-C and eGFR (Table 4). HTGL was positively correlated with ferritin levels (Table 4). TyG index was positively correlated with WC, TG, FPG, HbA1c, HOMA-IR, ferritin, WBC count, and CRP levels, while negatively corelated with LPL and HDL-C (Table 4).

These findings suggest that circulating LPL and HTGL levels are more affected by insulin resistance in NGT than in IFG.

### 3.5. Multiple Regression Analysis of the Predictors of the LPL, GPIHBP1, and HTGL Levels in the Peripheral Bloodstream

Based on the multiple regression analysis, among 207 participants, the circulating LPL level was independently affected by BMI, GPIHBP1, FPG, fasting insulin, HDL-C, CRP, and ferritin levels, and RBC count (Table 5). The circulating GPIHBP1 level was independently affected by age, eGFR, and LPL, HTGL, and FPG levels, and RBC count (Table 5). The circulating HTGL level was independently affected by WC and GPIHBP1 and LDL-C levels (Table 5). Among 153 participants classified as NGT, the circulating LPL level was independently affected by GPIHBP1, HFL-C, fasting insulin, ferritin, and CRP levels (Table 5). The circulating GPIHBP1 level was independently affected by LPL, HDL-C, HbA1c, eGFR, and RBC count (Table 5). The circulating HTGL levels were independently affected by GPIHBP1, HDL-C, and LDL-C levels (Table 5). GPIHBP1 is an anchor protein of LPL; however, the LPL and GPIHBP1 concentrations in the peripheral bloodstream are regulated by different mechanisms. In addition, the circulating HTGL level is regulated in a manner that promotes arteriosclerosis.

## 4. Discussion

This study showed that reciprocal fluctuations in LPL, GPIHBP1, and HTGL levels in the peripheral bloodstream are correlated with insulin resistance. Among middle-aged healthy and prediabetic Japanese individuals, elevated insulin resistance leads to decreased LPL levels and increased HTGL levels in the peripheral bloodstream. Circulating levels of LPL and HTGL are more affected by insulin resistance in females than in males. Circulating GPIHBP1 levels are affected in a compensatory manner in response to a decrease in circulating LPL levels correlated with insulin resistance. The correlation between reciprocal fluctuations in LPL, GPIHBP1, and HTGL and insulin resistance was evident under physiological conditions and was attenuated in IFG.

The role of insulin in regulating circulating LPL levels in the peripheral bloodstream is yet to be elucidated. Insulin administration promotes LPL production in vivo [9] and in vitro [36,37]. Miyashita et al. proposed that decreased circulating LPL levels in patients with T2DM are caused by reduced insulin secretion and its action [9]. By contrast, patients with metabolic diseases, diabetes, and CVD have low circulating LPL levels [4,5,6,7,8,9,10,11,12,13,14,15]. Metabolic diseases, diabetes, and CVD progress with increasing insulin resistance [4,5,6,7,8,9,10,11,12,13,14]. Our investigation supported the latter. Higher fasting insulin levels attributed to increased insulin resistance were correlated with lower LPL levels in the peripheral bloodstream among participants classified as NGT. Consistent with our results, Hanyu et al. showed that circulating LPL mass reflected insulin resistance in NGT, IGT, and T2DM [38,39]. Based on a previous report, insulin secretion decreases with age in Japanese individuals [27]. Similarly, in this study, age was negatively correlated with HOMA-β scores and was positively correlated with FPG and HbA1c levels (Supplementary Table S2). However, fasting serum insulin levels were not correlated with age (Supplementary Table S2). This result could be explained by the fact that this study included participants whose basal insulin secretion had begun to decline. In fact, in this study, HOMA-β score was reduced in the IFG group compared to the NGT group. The increase in the proportion of middle-aged Japanese individuals with T2DM is largely attributed to a decline in insulin secretion ability [27,40]. Based on these facts, patients with T2DM were excluded from this study. Insulin resistance is correlated with a high BMI, large WC, increased TG level, and decreased HDL-C level [27,41,42,43]. Consistent with these reports, this study revealed that BMI, WC, and TG were positively correlated with HOMA-IR scores. Meanwhile, the HDL-C level was negatively correlated with HOMA-IR scores (Supplementary Table S2). The circulating LPL levels were negatively correlated with WC, TG levels, and BMI, and were positively correlated with HDL-C levels. In recent years, the TyG index has attracted attention as an indicator of insulin resistance. Insulin concentration is not used to calculate the TyG index [28,29]. In this study, the TyG index was positively correlated with age, HOMA-IR score, WBC count, BMI, WC, and TG, LDL-C, fasting insulin, FPG, HbA1c, ferritin, and CRP levels. Both ferritin and CRP levels reflected systemic inflammation and insulin resistance [44,45,46]. Further, TyG index was negatively correlated with LPL and HDL-C levels. These data suggest that insulin resistance reduces circulating LPL levels in the peripheral bloodstream. According to a multiple regression analysis, circulating LPL levels were affected by FPG, fasting insulin, ferritin, HDL-C, CRP, and GPIHBP1. These data indicate that higher levels of circulating insulin due to insulin resistance suppress circulating LPL levels. We have previously reported the positive correlation between serum adiponectin and LPL concentrations [5]. Adiponectin is an inducer of LPL secretion and has been reported to be a factor reflecting insulin resistance [5,47,48]. To elucidate the regulatory mechanism of circulating LPL levels, it is necessary to consider the effects of both insulin and adiponectin. The correlation between circulating levels of LPL and insulin was evident under physiological conditions and was attenuated in IFG. Insulin resistance has a greater effect on circulating LPL levels in females than in males.

This study revealed that the TyG index had a good correlation with indices reflecting insulin resistance, such as BMI, WC, HOMA-IR score, WBC count, and fasting insulin, as well as FPG, HbA1c, HDL-C, TG, ferritin, and CRP levels. The HOMA-IR score was correlated with the HTGL levels, but not with the LDL-C levels (Supplementary Table S2). Meanwhile, the TyG index was correlated with the LDL-C levels, but not with the HTGL levels. The LDL-C level was correlated with HTGL, TG, and FPG levels, but not with fasting insulin levels (Table 2 and Supplementary Table S2). Based on these data, the TyG index is a useful indicator of insulin resistance even in patients with reduced insulin secretory ability because it does not use insulin as a variable.

Based on a previous study, circulating HTGL levels were correlated with small-dense LDL-C levels and remnant lipoprotein cholesterol levels. Moreover, high HTGL levels were found to increase the risk of CVD in middle-aged Japanese individuals [15]. Similarly, our previous report showed that circulating HTGL levels were positively correlated with LDL-C levels in young Japanese individuals [22]. These data indicate that circulating HTGL levels reflect LDL-C metabolism in both young and middle-aged Japanese individuals. Additionally, in this study, circulating HTGL levels had a good correlation with indices reflecting insulin resistance. Based on these results, individuals with a higher insulin resistance presented with elevated circulating HTGL levels. Like LPL, circulating HTGL levels are more affected by insulin resistance in females than in males. The correlation between circulating levels of HTGL and insulin was evident under physiological conditions and was attenuated in IFG. Further, these results indicate that reciprocal fluctuations in LPL and HTGL levels affect LDL-C metabolism in the peripheral bloodstream via their correlation with insulin resistance. This hypothesis is consistent with previous reports. Circulating levels of LPL were positively correlated with adiponectin levels [4,5,49,50], and negatively correlated with HTGL levels [50,51]. Adiponectin plays an important role in regulating the circulating level of LPL and HTGL in peripheral bloodstream.

This study showed that across the 207 participants, circulating GPIHBP1 levels were positively correlated with LPL and HTGL levels. The correlation between GPIHBP1 and LPL was evident under physiological conditions and was attenuated in IFG. No statistically significant differences were observed in GPIHBP1 between the NGT and IFG groups. In addition, circulating GPIHBP1 levels were independently affected by age, RBC count, LPL, HTGL, FPG levels and eGFR. In total 207 participants, the circulating LPL levels were positively correlated with HDL-C levels and negatively correlated with TG levels. Meanwhile, the circulating GPIHBP1 levels were correlated with neither HDL-C nor TG levels. By contrast, according to a previous report, circulating LPL levels were positively correlated with GPIHBP1 levels, and both LPL and GPIHBP1 levels were negatively correlated with TG levels in young Japanese [22]. This finding suggests that the correlation between circulating levels of GPIHBP1 and TG in the peripheral bloodstream differs between young and middle-aged Japanese individuals. Based on the multiple regression analysis, circulating GPIHBP1 levels were independently affected by eGFR. These findings suggest that in middle-aged Japanese individuals classified as NGT or IFG, circulating GPIHBP1 levels might be determined by renal function rather than insulin resistance. The positive correlation between circulating levels of HTGL and GPIHBP1 could be explained by an increase in GPIHBP1 levels, which may compensate for the decrease in LPL levels attributed to increased insulin resistance. Further studies are needed to clarify the effect of adiponectin on GPIHBP1.

This study included individuals who are treated with statins and antihypertensive drugs. Some reports have revealed that the administration of statins and antihypertensive drugs significantly increases circulating LPL levels. However, some studies have shown no significant difference in circulating LPL levels between patients who are receiving statins and antihypertensive drugs and those who are not [52]. This study found that circulating LPL levels were more likely to increase with statin administration. The statin-treated group had a greater BMI, larger WC, and higher HbA1c than the non-statin-treated group. Therefore, patients receiving statins had a higher insulin resistance. A high insulin resistance affected circulating levels of LPL and GPIHBP1 in the statin-treated groups. Therefore, there was no significant difference in the LPL levels between the statin-treated and non-treated groups. Similarly, the antihypertensive drug-treated group had a higher GPIHBP1, BMI, WC, FPG, and TyG index. Ultimately, to properly evaluate the effects of therapeutic drugs such as statins and antihypertensive drugs on circulating LPL levels, comparisons before and after administration are necessary.

This study had several limitations. First, it was a cross-sectional study, and it included a relatively small number of participants. Thus, large-scale prospective studies should be performed to confirm our hypothesis. Second, the study-design precludes examination of causality for the relationship between insulin resistance and LPL, HTGL, and GPIHBP1. Third, the activity of LPL and HTGL was not assessed. To validate the correlation between the lipolytic ability of circulating LPL and GPIHBP1 levels, the activity of LPL and HTGL should be investigated. However, currently, there is no available method that can measure LPL and HTGL activity in the peripheral blood under physiological conditions.

## 5. Conclusions

This study showed that reciprocal fluctuations in LPL and HTGL levels in the peripheral bloodstream are correlated with insulin resistance in middle-aged Japanese individuals. The correlation between reciprocal fluctuations in LPL, GPIHBP1, and HTGL and insulin resistance was evident under physiological conditions and was attenuated in IFG. Further, circulating GPIHBP1 levels may be affected by renal function and in a compensatory manner in response to a decrease in circulating LPL levels correlated with insulin resistance. The effect of insulin resistance on circulating LPL and HTGL levels is greater in females than in males.

## Figures and Tables

**Table 1:** Characteristics of 207 participants.

	Total (n = 207)	Male (n = 100)	Female (n = 107)	*p*
Age (years)	58 (49–64)	57 (50–64)	58 (49–64)	0.929
NGT/IFG	153/54	70/30	83/24	0.215
Statin (+/−)	64/143	28/72	36/71	0.380
Antihypertensive drugs (+/−)	110/97	70/30	44/68	<0.001 **
Current smoking (+/−)	28/179	23/77	7/105	<0.001 **
Exercise habits (+/−)	103/104	51/49	52/60	0.506
BMI (kg/m^2^)	23.6 (21.5–25.5)	24.5 (22.2–26.4)	22.4 (20.2–24.7)	<0.001 **
Waist circumference (cm)	84.5 (79.0–90.0)	88.0 (82.5–93.0)	810 (74.0–87.1)	<0.001 **
SBP (mmHg)	123 (114–132)	123 (116–133)	123 (113–131)	0.254
DBP (mmHg)	79 (70–86)	83 (74–88)	75 (67–82)	<0.001 **
LPL (ng/mL)	77.9 (61.8–97.5)	73.0 (58.3–88.2)	85.4 (68.4–108.6)	<0.001 **
GPIHBP1 (pg/mL)	853.5 (700.4–1013.7)	891.7 (696.0–1178.5)	824.0 (700.4–964.4)	0.035 *
HTGL (ng/mL)	46.6 (33.0–60.0)	46.9 (32.8–59.8)	46.4 (34.6–60.6)	0.897
TC (mg/dL)	210 (193–236)	204 (184–227)	219 (199–243)	0.002 **
HDL-C (mg/dL)	66 (53–77)	56 (49–68)	70 (58–85)	<0.001 **
TG (mg/dL)	102 (69–136)	120 (92–159)	88 (60–121)	<0.001 **
LDL-C (mg/dL)	121 (97–141)	121 (95–136)	122 (99–145)	0.207
HbA1c (%)	5.8 (5.5–6.1)	5.8 (5.6–6.1)	5.8 (5.5–6.0)	0.657
FPG (mg/dL)	103 (97–110)	105 (100–111)	100 (95–108)	0.002 **
Insulin (µU/mL)	6.1 (4.3–8.8)	6.3 (4.4–9.1)	6.0 (4.2–8.4)	0.718
HOMA-β	57.4 (38.0–80.0)	56.1 (35.3–79.9)	57.8 (39.5–81.0)	0.227
HOMA-IR	1.6 (1.1–2.3)	1.8 (1.1–2.4)	1.5 (1.0–2.2)	0.937
TyG index	8.61 (8.19–8.93)	8.74 (8.44–9.05)	8.38 (8.00–8.80)	<0.001 **
Ferritin (ng/mL)	109.0 (64.1–190.7)	153.5 (92.3–252.4)	77.6 (33.2–125.9)	<0.001 **
RBC (×10^4^/µL)	461 (439–503)	500 (455–522)	446 (427–470)	<0.001 **
WBC (/µL)	5200 (4380–6173)	5680 (4480–6760)	4930 (4350–5870)	<0.001 **
Plt (×10^4^/µL)	23.2 (20.0–26.3)	23.1 (19.5–26.1)	23.3 (21.1–26.4)	0.369
CRP (mg/dL)	0.05 (0.05–0.13)	0.06 (0.05–0.15)	0.05 (0.05–0.1)	0.03 *
eGFR (mL/min/1.73 m^2^)	74 (64–83)	70 (61–82)	77 (69–83)	0.017 *

Data are shown as medians (first quartile–third quartile). NGT: normal glucose tolerance; IFG: impaired fasting glucose; BMI: body mass index; SBP: systolic blood pressure; DBP: diastolic blood pressure; LPL: lipoprotein lipase; GPIHBP1: glycosylphosphatidylinositol-anchored HDL binding protein 1; HTGL: hepatic triglyceride lipase; TC: total cholesterol; HDL-C: high-density lipoprotein cholesterol; TG: triglyceride; LDL-C: low-density lipoprotein cholesterol; FPG: fasting plasma glucose; HbA1c: hemoglobin A1c; HOMA-IR: Homeostasis Model Assessment of Insulin Resistance; HOMA-β: Homeostasis Model Assessment of Beta-cell function; TyG index: triglyceride glucose index; RBC: red blood cell count; WBC: white blood cell count; Plt: platelet count; CRP: C reactive protein; eGFR: estimated glomerular filtration rate. Significant between-groups differences were identified using the Mann–Whitney U test. Statistical significance was set at a *p*-value of < 0.05. *: *p* < 0.05; **: *p* < 0.01. Blue indicates lower values for males compared to females, red indicates higher values for males compared to females.

**Table 2:** Correlations between LPL; HTGL, GPIHBP1, and indicatiors of insulin resistance with clinical and laboratory parameters.

		LPL (ng/mL)			GPIHBP1 (pg/mL)			HTGL (ng/mL)			TyG Index		
		Total	Male	Female	Total	Male	Female	Total	Male	Female	Total	Male	Female
		n = 207	n = 100	n = 107	n = 207	n = 100	n = 107	n = 207	n = 100	n = 107	n = 207	n = 100	n = 107
Age	*r*	0.071	0.031	0.095	0.136	−0.025	**0.304**	0.064	0.031	0.101	**0.219**	0.049	**0.392**
	*p*	0.313	0.757	0.329	0.051	0.806	0.001 **	0.358	0.763	0.301	0.002 **	0.628	<0.001 **
BMI (kg/m^2^)	*r*	**−0.311**	**−0.309**	**−0.192**	0.084	0.055	0.054	**0.155**	0.103	**0.206**	**0.467**	**0.403**	**0.39**
	*p*	<0.001 **	0.002 **	0.048 *	0.231	0.587	0.578	0.026 *	0.308	0.033 *	<0.001 **	<0.001 **	<0.001 **
Waist circumference (cm)	*r*	**−0.3**	**−0.281**	−0.167	0.123	0.057	0.127	**0.203**	0.097	**0.301**	**0.5**	**0.378**	**0.467**
	*p*	<0.001 **	0.005 **	0.086	0.077	0.576	0.192	0.003 **	0.337	0.002 **	<0.001 **	<0.001 **	<0.001 **
LPL (ng/mL)	*r*	1.000	1.000	1.000	**0.244**	**0.313**	**0.245**	−0.114	−0.090	−0.125	**−0.329**	**−0.388**	−0.157
	*p*				<0.001 **	0.002 **	0.011 *	0.102	0.372	0.200	<0.001 **	<0.001 **	0.106
GPIHBP1 (pg/mL)	*r*	**0.244**	**0.313**	**0.245**	1.000	1.000	1.000	**0.146**	**0.228**	0.070	0.035	−0.100	0.092
	*p*	<0.001 **	0.002 **	0.011 *				0.036 *	0.023 *	0.474	0.616	0.324	0.344
HTGL (ng/mL)	*r*	−0.114	−0.090	−0.125	**0.146**	**0.228**	0.070	1.000	1.000	1.000	0.087	0.125	0.043
	*p*	0.102	0.372	0.200	0.036 *	0.023 *	0.474				0.215	0.216	0.660
TC (mg/dL)	*r*	0.117	−0.049	0.174	0.022	−0.031	0.158	0.105	**0.219**	0.028	0.132	0.155	**0.272**
	*p*	0.092	0.631	0.073	0.750	0.760	0.105	0.133	0.029 *	0.778	0.059	0.123	0.005 **
HDL-C (mg/dL)	*r*	**0.325**	**0.276**	**0.238**	−0.094	0.028	−0.128	**−0.163**	−0.067	**−0.261**	**−0.523**	**−0.466**	**−0.425**
	*p*	<0.001 **	0.005 **	0.014 *	0.178	0.785	0.190	0.019 *	0.508	0.007 **	<0.001 **	<0.001 **	<0.001 **
TG (mg/dL)	*r*	**−0.312**	**−0.379**	−0.137	0.042	−0.097	0.133	0.079	0.100	0.051	**0.982**	**0.984**	**0.981**
	*p*	<0.001 **	<0.001 **	0.158	0.546	0.337	0.172	0.261	0.322	0.598	<0.001 **	<0.001 **	<0.001 **
LDL-C (mg/dL)	*r*	−0.005	−0.088	0.038	0.063	−0.044	**0.21**	**0.233**	**0.225**	**0.249**	**0.251**	0.187	**0.38**
	*p*	0.945	0.386	0.698	0.363	0.666	0.030 *	<0.001 **	0.025 *	0.01 **	<0.001 **	0.062	<0.001 **
HbA1c (%)	*r*	−0.065	−0.005	−0.082	−0.050	−0.039	−0.089	−0.004	0.057	−0.062	**0.253**	0.125	**0.366**
	*p*	0.354	0.964	0.402	0.472	0.702	0.362	0.959	0.570	0.523	<0.001 **	0.215	<0.001 **
FPG (mg/dL)	*r*	**−0.233**	−0.117	**−0.215**	−0.012	0.023	−0.116	0.064	0.095	0.025	**0.476**	**0.256**	**0.592**
	*p*	<0.001 **	0.248	0.026 *	0.865	0.822	0.235	0.362	0.347	0.796	<0.001 **	<0.001 **	<0.001 **
Insulin (µU/mL)	*r*	**−0.268**	−0.094	**−0.405**	−0.019	0.078	−0.155	**0.159**	0.040	**0.274**	**0.22**	**0.263**	0.178
	*p*	<0.001 **	0.352	<0.001 **	0.785	0.443	0.112	0.022 *	0.695	0.004 **	0.001 **	0.008 **	0.066
HOMA-β	*r*	−0.135	−0.049	**−0.237**	0.023	0.102	−0.038	0.113	−0.010	**0.203**	−0.009	0.158	−0.139
	*p*	0.053	0.632	0.014 *	0.738	0.314	0.696	0.106	0.919	0.036 *	0.896	0.117	0.154
HOMA-IR	*r*	**−0.306**	−0.118	**−0.444**	−0.031	0.067	−0.171	**0.159**	0.047	**0.257**	**0.283**	**0.286**	**0.27**
	*p*	<0.001 **	0.244	<0.001 **	0.653	0.506	0.078	0.022 *	0.645	0.008 **	<0.001 **	0.004 **	0.005 **
TyG index	*r*	**−0.329**	**−0.0388**	−0.157	0.035	−0.100	0.092	0.087	0.125	0.043	1.000	1.000	1.000
	*p*	<0.001 **	<0.001 **	0.106	0.616	0.324	0.344	0.215	0.216	0.660			
Ferritin (ng/mL)	*r*	**−0.347**	**−0.306**	**−0.22**	0.048	0.017	0.021	**0.214**	0.170	**0.284**	**0.273**	**0.329**	0.000
	*p*	<0.001 **	0.002 **	0.023 *	0.495	0.867	0.832	0.002 **	0.091	0.003 **	<0.001 **	<0.001 **	0.999
RBC (×10^4^/µL)	*r*	**−0.203**	−0.111	−0.017	−0.017	−0.037	−0.100	0.063	0.093	0.054	**0.256**	0.101	0.103
	*p*	0.003 **	0.273	0.861	0.813	0.716	0.306	0.364	0.356	0.580	<0.001 **	0.317	0.291
WBC (/µL)	*r*	**−0.206**	−0.024	**−0.0304**	0.002	0.004	−0.046	0.000	0.003	0.013	**0.279**	**0.198**	**0.246**
	*p*	0.003 **	0.815	0.001 *	0.974	0.968	0.638	0.995	0.976	0.893	<0.001 **	0.049 *	0.011 *
Plt (×10^4^/µL)	*r*	−0.049	−0.102	−0.075	−0.069	−0.113	0.006	0.134	0.156	0.115	0.085	0.087	0.151
	*p*	0.484	0.313	0.441	0.326	0.262	0.951	0.053	0.122	0.238	0.221	0.388	0.121
CRP (mg/dL)	*r*	**−0.289**	**−0.199**	**−0.31**	0.049	0.017	0.047	0.123	0.021	**0.233**	**0.231**	**0.248**	0.130
	*p*	<0.001 **	0.049 *	0.001 **	0.489	0.864	0.638	0.081	0.837	0.017 *	<0.001 **	0.014 *	0.187
eGFR (mL/min/1.73 m^2^)	*r*	0.042	−0.122	0.075	**−0.263**	**−0.227**	**−0.335**	−0.118	**−0.206**	−0.037	**−0.178**	−0.022	−0.178
	*p*	0.550	0.227	0.444	<0.001 **	0.023 *	<0.001 **	0.091	0.039 *	0.706	0.010 *	0.831	0.067

BMI: body mass index; SBP: systolic blood pressure; DBP: diastolic blood pressure; LPL: lipoprotein lipase; GPIHBP1: glycosylphosphatidylinositol-anchored HDL binding protein 1; HTGL: hepatic triglyceride lipase; TC: total cholesterol; HDL-C: high-density lipoprotein cholesterol; TG: triglyceride; LDL-C: low-density lipoprotein cholesterol; FPG: fasting plasma glucose; HbA1c: hemoglobin A1c; HOMA-IR: Homeostasis Model Assessment of Insulin Resistance; HOMA-β: Homeostasis Model Assessment of Beta-cell function; TyG index: triglyceride glucose index; RBC: red blood cell count; WBC: white blood cell count; Plt: platelet count; CRP: C reactive protein; eGFR: estimated glomerular filtration rate. Spearman correlation analyses were conducted for total 207 participants, 100 males, and 107 females. Statistical significance was set at a *p*-value of < 0.05. *: *p* < 0.05; **: *p* < 0.01. Blue indicates negative correlation, red indicates positive correlation.

**Table 3:** Comparison of LPL, GPIHBP1, and HTGL and clinical and laboratory parameters between NGT and IFG.

	Total			Male			Female			NGT	IFG
										Male vs. Female	Male vs. Female
	NGT (n = 153, 73.9%)	IFG (n = 54, 26.1%)	*p*	NGT (n = 70, 70%)	IFG (n = 30, 30%)	*p*	NGT (n = 83, 77.6%)	IFG (n = 24, 22.4%)	*p*	*p*	*p*
Age (years)	57 (49–63)	60 (51–67)	0.091	59 (50–64)	56 (51–63)	0.670	57 (46–63)	64 (52–69)	0.008 **	0.261	0.036 *
Statin (+/−)	46/107	18/36	0.655	19/51	9/21	0.771	27/56	9/15	0.650	0.469	0.561
Antihypertensive drugs (+/−)	79/74	31/23	0.465	52/18	18/12	0.153	27/56	18/6	0.008 **	<0.001 **	0.245
Current smoking (+/−)	17/136	11/43	0.087	13/57	10/20	0.101	4/79	1/23	0.894	0.007 **	0.008 **
Exercise habits (+/−)	72/81	31/23	0.191	33/37	18/12	0.239	39/44	13/11	0.534	0.985	0.667
BMI (kg/m^2^)	23.1 (21.1–25.0)	24.8 (22.7–27.6)	<0.001 **	23.9 (21.8–25.9)	24.9 (23.5–27.9)	0.091	21.6 (19.8–23.8)	24.4 (22.5–27.3)	0.004 **	<0.001 **	0.317
Waist circumference (cm)	83.5 (76.5–89.5)	87.8 (82.9–9.4)	<0.001 **	88.1 (82.2–92.2)	88.3 (82.5–95.8)	0.364	80.0 (73.5–85.5)	86.0 (83.0–92.0)	0.002 **	<0.001 **	0.268
SBP (mmHg)	123 (114–132)	125 (116–138)	0.056	127 (117–133)	122 (109–134)	0.454	122 (111–129)	130 (123–144)	0.001 **	0.008 **	0.039 *
DBP (mmHg)	77 (69–86)	81 (72–88)	0.129	84 (75–88)	83 (72–88)	0.937	73 (66–81)	79 (71–86)	0.111	<0.001 **	0.261
LPL (ng/mL)	80.7 (66.2–101.5)	73.2 (55.3–88.7)	0.041 *	73.3 (61.3–86.6)	72.9 (53.0–89.2)	0.778	87.9 (73.1–114)	74.4 (58.7–89.0)	0.041 *	<0.001 **	0.48
GPIHBP1 (pg/mL)	857.6 (702.2–993.7)	840.7 (689.3–1094.7)	0.631	885.9 (690.3–1145.2)	910.6 (716.9–1199.7)	0.463	848 (712.6–957.8)	770.3 (660.6–1046.1)	0.911	0.069	0.276
HTGL (ng/mL)	45.8 (33.8–58.6)	50.1 (32.9–73.0)	0.176	46.5 (32.9–57.1)	48.4 (32.3–72.0)	0.179	44.7 (34.6–59.2)	50.9 (33.4–74.4)	0.581	0.561	0.721
TC (mg/dL)	207 (188–230)	221 (201–242.3)	0.053	202 (179–222.5)	213 (197.5–242.5)	0.035 *	215 (195–243)	227 (211–244.5)	0.328	<0.001 **	0.334
HDL-C (mg/dL)	67 (54–82)	56 (51–68)	0.004 *	57 (49–72.3)	53 (48–67)	0.286	73 (64–86)	59 (53.5–70.8)	0.017 *	<0.001 **	0.11
TG (mg/dL)	97 (65–132)	119 (88–147.3)	0.060	112 (85.5–163.3)	128.5 (97.5–157)	0.427	81 (55–111)	112.5 (79–138)	0.025 *	<0.001 **	0.12
LDL-C (mg/dL)	114 (95–136)	134.5 (119.8–152.3)	0.004 **	114 (92.5–127.3)	134.5 (120–152.3)	0.002 **	113 (97–143)	135.5 (115.3–157.0)	0.039 *	0.126	0.566
HbA1c (%)	5.7 (5.5–6.0)	6.0 (5.8–6.4)	<0.001 **	5.7 (5.5–6.0)	6.1 (5.8–6.4)	0.002 **	5.8 (5.4–6.0)	5.9 (5.8–6.4)	0.006 **	0.906	0.9
FPG (mg/dL)	100 (95–104)	116 (112–123)	<0.001 **	103 (97–107)	116 (112–122)	<0.001 **	98 (93–102)	116 (112–123)	<0.001 **	<0.001 **	0.867
Insulin (µU/mL)	6.0 (4.1–8.5)	7.0 (5.5–9.2)	0.706	6.1 (4.0–9.0)	7.6 (5.6–12.4)	0.608	5.7 (4.1–7.9)	6.8 (5.2–8.5)	0.361	0.574	0.241
HOMA-β	60 (38.9–84.8)	48.4 (35.4–62.3)	0.008 **	60.9 (34.7–81.9)	50.5 (36.4–64.5)	0.125	60.0 (40.5–88.9)	45.7 (33.1–57.3)	0.023 *	0.231	0.443
HOMA-IR	1.4 (1.0–2.1)	2.0 (1.6–2.7)	0.298	1.5 (1.0–2.3)	2.2 (1.7–3.5)	0.144	1.3 (1.0–2.0)	1.9 (1.6–2.5)	0.995	0.762	0.169
TyG index	8.50 (8.04–8.81)	8.87 (8.52–9.10)	<0.001 **	8.65 (8.43–8.98)	8.90 (8.61–9.16)	0.053	8.27 (7.92–8.63)	8.83 (8.41–9.00)	<0.001 **	<0.001 **	0.165
Feritin (ng/mL)	109 (68.2–182.1)	108.4 (44.9–195.7)	0.467	165 (94.3–261.2)	125.5 (63.9–231.4)	0.108	84.1 (47.9–125.1)	76.3 (27.2–158.8)	0.866	<0.001 **	0.053
RBC (×10^4^/µL)	459 (434–499)	481 (441–510)	0.108	495 (451–520)	504 (481–531)	0.065	445 (430–464)	449 (414–474)	0.620	<0.001 **	<0.001 **
WBC (/µL)	5080 (4310–6060)	5805 (4813–6530)	<0.011 *	5465 (4293–6723)	5805 (5080–6943)	0.098	4830 (4320–5720)	5555 (5080–6943)	0.103	0.007 **	0.063
Plt (×10^4^/µL)	23.1 (19.8–26.3)	23.4 (21.7–26.4)	0.291	21.7 (19–26.1)	23.6 (21.1–26.5)	0.313	23.3 (20.4–26.4)	23.4 (22.5–26.3)	0.577	0.336	0.783
CRP (mg/dL)	0.05 (0.05–0.11)	0.06 (0.05–0.21)	0.206	0.06 (0.05–0.14)	0.06 (0.05–0.23)	0.640	0.05 (0.05–0.07)	0.06 (0.05–0.19)	0.640	0.03 *	0.562
eGFR (mL/min/1.73 m^2^)	74 (65–83)	73 (63–84)	0.948	70 (60–82)	68.5 (62.8–79.8)	0.416	77 (70–83)	73.5 (62.3–84.5)	0.492	0.012 *	0.752

Data are shown as median (first quartile–third quartile). NGT: normal glucose tolerance; IFG: impaired fasting glucose; BMI: body mass index; WC: waist circumference; SBP: systolic blood pressure; DBP: diastolic blood pressure; LPL: lipoprotein lipase; GPIHBP1: glycosylphosphatidylinositol-anchored HDL binding protein 1; HTGL: hepatic triglyceride lipase; TC: total cholesterol; HDL-C: high-density lipoprotein cholesterol; TG: triglyceride; LDL-C: low-density lipoprotein cholesterol; FPG: fasting plasma glucose; HbA1c: hemoglobin A1c; HOMA-IR: Homeostasis Model Assessment of Insulin Resistance; HOMA-β: Homeostasis Model Assessment of Beta-cell function; TyG index: triglyceride glucose index; CRP: C reactive protein; eGFR: estimated glomerular filtration rate. Significant between-groups differences were identified using the Mann–Whitney U test. Statistical significance was set at a *p*-value of < 0.05. *: *p* < 0.05; **: *p* < 0.01. Blue text indicates lower values in the IFG group compared to the NGT group or lower values in the male group compared to the female group, and red text indicates higher values in the IFG group compared to the NGT group or higher values in the male group compared to the female group.

**Table 4:** Correlation of LPL, GPIHBP1, and HTGL with clinical and laboratory parameters in 153 participants clasified with NGT and 54 participants classified with IFG.

		LPL (ng/mL)		GPIHBP1 (pg/mL)		HTGL (ng/mL)		TyG Index	
		NGT	IFG	NGT	IFG	NGT	IFG	NGT	IFG
		n = 153	n = 54	n = 153	n = 54	n = 153	n = 54	n = 153	n = 54
Age (years)	*r*	0.124	0.024	**0.171**	0.053	0.004	0.241	**0.218**	0.165
	*p*	0.125	0.863	0.035 *	0.703	0.956	0.080	0.007 **	0.232
BMI (kg/m^2^)	*r*	**−0.289**	−0.219	0.085	0.125	**0.185**	−0.025	**0.453**	0.260
	*p*	<0.001 **	0.112	0.294	0.368	0.022 *	0.858	<0.001 **	0.058
Waist circumference (cm)	*r*	**−0.289**	−0.204	0.083	0.250	**0.209**	0.140	**0.46**	**0.447**
	*p*	<0.001 **	0.140	0.307	0.068	0.01 **	0.313	<0.001 **	<0.001 **
LPL (ng/mL)	*r*	1.000	1.000	**0.239**	0.258	−0.100	−0.115	**−0.269**	**−0.335**
	*p*			0.003 **	0.059	0.218	0.409	<0.001 **	0.013 *
GPIHBP1 (pg/mL)	*r*	**0.239**	0.258	1.000	1.000	0.148	0.161	−0.030	0.204
	*p*	0.003 **	0.059			0.068	0.246	0.715	0.139
HTGL (ng/mL)	*r*	−0.100	−0.115	0.148	0.161	1.000	1.000	0.021	0.123
	*p*	0.218	0.409	0.068	0.246			0.799	0.375
TC (mg/dL)	*r*	**0.221**	−0.097	0.112	−0.211	0.078	0.071	0.070	0.154
	*p*	0.006 **	0.487	0.169	0.125	0.339	0.611	0.390	0.267
HDL-C (mg/dL)	*r*	**0.377**	0.033	0.003	**−0.364**	**−0.203**	0.000	**−0.511**	**−0.426**
	*p*	<0.001 **	0.813	0.969	0.007 **	0.012 *	0.997	<0.001 **	<0.001 **
TG (mg/dL)	*r*	**−0.265**	**−0.338**	−0.021	0.203	0.026	0.142	**0.994**	**0.984**
	*p*	<0.001 **	0.013 *	0.793	0.141	0.746	0.305	<0.001 **	<0.001 **
LDL-C (mg/dL)	*r*	0.067	−0.049	0.120	−0.105	**0.233**	0.125	**0.194**	0.104
	*p*	0.413	0.727	0.139	0.450	0.004 **	0.368	0.016 *	0.453
HbA1c (%)	*r*	0.077	−0.236	−0.086	0.062	−0.042	−0.035	0.155	**0.274**
	*p*	0.341	0.086	0.291	0.656	0.609	0.802	0.056	0.045 *
FPG (mg/dL)	*r*	**−0.199**	−0.139	−0.014	−0.044	−0.014	0.115	**0.441**	**0.32**
	*p*	0.014 *	0.315	0.860	0.754	0.867	0.406	<0.001 **	0.018 *
Insulin (µU/mL)	*r*	**−0.306**	−0.042	−0.091	0.215	0.138	0.177	**0.177**	0.220
	*p*	<0.001 **	0.761	0.266	0.119	0.089	0.199	0.028 *	0.110
HOMA-β	*r*	**−0.26**	0.005	−0.081	0.240	0.132	0.139	0.029	0.136
	*p*	0.001 **	0.971	0.318	0.080	0.103	0.316	0.718	0.328
HOMA-IR	*r*	**−0.321**	−0.062	−0.093	0.182	0.133	0.196	**0.217**	**0.273**
	*p*	<0.001 **	0.654	0.254	0.189	0.100	0.155	0.007 **	0.046 *
TyG index	*r*	**−0.269**	**−0.335**	−0.030	0.204	0.021	0.123	1.000	1.000
	*p*	0.001 **	0.013 *	0.715	0.139	0.799	0.375		
Ferritin (ng/mL)	*r*	**−0.393**	**−0.28**	0.013	0.129	0.132	**0.405**	**0.251**	**0.398**
	*p*	<0.001 **	0.04 *	0.874	0.353	0.105	0.002 **	0.002 **	0.003 **
RBC (×10^4^/µL)	*r*	**−0.285**	0.048	−0.059	0.116	0.069	0.043	**0.245**	0.169
	*p*	<0.001 **	0.730	0.472	0.406	0.398	0.760	0.002 **	0.221
WBC (/µL)	*r*	**−0.215**	−0.066	−0.075	0.196	0.020	−0.087	**0.187**	**0.394**
	*p*	0.007 **	0.634	0.357	0.154	0.808	0.532	0.021 *	0.003 **
Plt (×10^4^/µL)	*r*	−0.047	0.041	−0.106	0.068	0.123	0.080	0.070	0.031
	*p*	0.563	0.768	0.194	0.626	0.129	0.565	0.389	0.823
CRP (mg/mL)	*r*	**−0.32**	−0.181	0.037	0.050	**0.174**	−0.008	0.150	**0.33**
	*p*	<0.001 **	0.192	0.652	0.720	0.034 *	0.954	0.069	0.015 *
eGFR (mL/min/1.73 m^2^)	*r*	0.027	−0.029	**−0.246**	**−0.311**	−0.156	−0.017	−0.158	−0.213
	*p*	0.740	0.836	0.002 **	0.022 *	0.055	0.903	0.050	0.123

NGT: normal glucose tolerance; IFG: impaired fasting glucose; BMI: body mass index; WC: waist circumference; LPL: lipoprotein lipase; GPIHBP1: glycosylphosphatidylinositol-anchored HDL binding protein 1; HTGL: hepatic triglyceride lipase; TC: total cholesterol; HDL-C: high-density lipoprotein cholesterol; TG: triglyceride; LDL-C: low-density lipoprotein cholesterol; FPG: fasting plasma glucose; HbA1c: hemoglobin A1c; HOMA-IR: Homeostasis Model Assessment of Insulin Resistance; HOMA-β: Homeostasis Model Assessment of Beta-cell function; TyG index: triglyceride glucose index; RBC: red blood cell count; WBC: white blood cell count; Plt: platelet count; CRP: C reactive protein; eGFR: estimated glomerular filtration rate. Spearman correlation analyses were conducted for 153 participants classified as NGT, and 54 participants classified as IFG. Statistical significance was set at a *p*-value of < 0.05. *: *p* < 0.05; **: *p* < 0.01. Blue indicates negative correlation, red indicates positive correlation.

**Table 5:** Multiple regression analysis of LPL, GPIIHBP1, HTGL and clinical laboratory parameters in 207 participants and 153 participants classified as NGT.

	LPL (ng/mL)				GPIHBP1 (pg/mL)				HTGL (ng/mL)			
	Total (n = 207)		NGT (n = 153)		Total (n = 207)		NGT (n = 153)		Total (n = 207)		NGT (n = 153)	
	*β*	*p*	*β*	*p*	*β*	*p*	*β*	*p*	*β*	*p*	*β*	*p*
Age (years)					−0.158	0.011 *						
BMI (kg/m^2^)	−0.206	0.004 **										
WC (cm)									0.140	0.042 *		
LPL (ng/mL)					0.253	<0.001 **	0.203	0.006 **				
GPIHBP1 (pg/mL)	0.223	<0.001 **	0.160	0.031 *					0.172	0.012 *	0.177	0.028 *
HTGL (ng/dL)					0.121	0.041 *						
HDL-C (mg/dL)	0.167	<0.021 *	0.245	0.002 **			−0.174	0.015 *			−0.159	0.049 *
LDL-C (mg/dL)									0.178	0.009 **	0.169	0.037 *
HbA1c (%)							−0.161	0.019 *				
FPG (mg/dL)	−0.132	0.039 *			−0.197	0.002 **						
Insulin (µU/mL)	−0.164	0.007 **	−0.193	0.007 **								
Ferritin (ng/mL)	−0.274	<0.001 **	−0.257	<0.001 **								
CRP (mg/dL)	0.135	0.038 *	−0.164	0.031 *								
RBC (×10^4^/µL)	−0.274	<0.001 **			−0.144	0.018 *	−0.156	0.031 *				
eGFR (mL/min/1.73 m^2^)					−0.503	<0.001 **	−0.520	<0.001 *				

BMI: body mass index; WC: waist circumference; LPL: lipoprotein lipase; GPIHBP1: glycosylphosphatidylinositol-anchored HDL binding protein 1; HTGL: hepatic triglyceride lipase; HDL-C: high-density lipoprotein cholesterol; LDL-C: low-density lipoprotein cholesterol; HbA1c: hemoglobin A1c: FPG: fasting plasma glucose; CRP: C-reactive protein; RBD: red blood cell count; eGFR: estimated glomerular filtration rate. A multiple regression analysis using the stepwise forward selection method was conducted on each variable to examine the relative contribution to circulating LPL, GPIHBP1, and HTGL levels. Statistical significance was set at a *p*-value of < 0.05. *: *p* < 0.05; **: *p* < 0.01.

## Data Availability

The datasets generated during and/or analyzed during the current study are available from the corresponding author on reasonable request. All data generated or analyzed during this study are included in this published article.

## Supplementary Materials

The following supporting information can be downloaded at: https://www.mdpi.com/article/10.3390/nu17111880/s1, Figure S1: Classification of glucose tolerance on 75 g OGTT, Table S1: Effects of statins and anti-hypertensive drugs on LPL, GPIHBP1, HTGL and other parameters in 207 participants; Table S2: Correlation of fasting insulin, FPG, HbA1c, HOMA-β, and HOMA-IR with other parameters in 207 participants.

## Abbreviations

BMI	Body mass index
CKD	Chronic kidney disease
CRP	C-reactive protein
CVD	Cardiovascular disease
DBP	Diastolic blood pressure
eGFR	Estimated glomerular filtration rate
ELISA	Enzyme-linked immunosorbent assay
FPG	Fasting plasma glucose
GPIHBP1	Glycosylphosphatidylinositol-anchored high-density lipoprotein-binding protein 1
HbA1c	Hemoglobin A1c
HDL-C	High-density lipoprotein cholesterol
HOMA-β	Homeostasis Model Assessment of β-Cell Function
HOMA-IR	Homeostasis Model Assessment of Insulin Resistance
HTGL	Hepatic triglyceride lipase
IFG	Impaired fasting glucose
LDL-C	Low-density lipoprotein cholesterol
LPL	Lipoprotein lipase
NGT	Normal glucose tolerance
RBC	Red blood cell
SBP	Systolic blood pressure
T2DM	Type II diabetes
TC	Total cholesterol
TG	Triglyceride
TyG index	Triglyceride–glucose index
WBC	White blood cell
WC	Waist circumference
