# Supplementary material for: Reciprocal Fluctuations in Lipoprotein Lipase, Glycosylphosphatidylinositol-Anchored High-Density Lipoprotein-Binding Protein 1, and Hepatic Triglyceride Lipase Levels in the Peripheral Bloodstream Are Correlated with Insulin Resistance
Source: Nutrients. 2025 May 30;17(11):1880. doi: 10.3390/nu17111880 (PMC12157957; doi:10.3390/nu17111880)
Supplement: Supplementary file 1 [file nutrients-17-01880-s001.zip › nutrients-3652406-supplementary.pdf]

Supplemental Table S1. Effects of statins and anti-hypertensive drugs on LPL, GPIHBP1, HTGL and clinical and laboratory parameters in 207 participants.

	Statin			Anti-hypertensive drugs		
	-	+	*p*	-	+	*p*
Age (years)	56 (48-62)	61 (54-67)	<0.001**	56 (46-63)	59 (53-64)	0.003**
Sex (men:/women:)	72/71	28/36	0.380	30/67	70/40	0.23
BMI (Kg/m^2)	22.7 (21.0-25.0)	24.3 (22.8-26.5)	0.009**	22.3 (20.7-24.4)	24.5 (22.1-26.4)	<0.001**
Waist circumference (cm)	82.5 (76.5-89.0)	88.0 (83.3-92.9)	<0.001**	82 (75.5-87)	88 (81.5-92.6)	<0.001**
SBP (mmHg)	124 (114-132)	123 (114-132)	0.897	122 (111-131)	126 (116-133)	0.012*
DBP (mmHg)	79 (71-87)	76 (67-85)	0.149	74 (66-83)	82 (74-87)	<0.001**
LPL (ng/mL)	76.6 (60.9-95.9)	83.2 (66.5-109.0)	0.058	78.9 (65.9-96.0)	77.0 (60.0-101.2)	0.259
GPIHBP1 (pg/mL)	824 (688.9-978.6)	934.5 (763.1-1091.4)	0.180	850 (691.8-971.2)	863.5 (703.6-1114.6)	0.027*
HTGL (ng/mL)	46.7 (32.5-59.4)	46.0 (36.3-64.6)	0.217	46.7 (34.6-57.3)	46.5 (32.9-63.0)	0.189
TC (mg/dL)	214 (195-241)	208 (188-224)	0.020*	215 (195-245)	207 (188-228)	0.003**
HDL-C (mg/dL)	67 (54-83)	61 (52-71)	0.048*	70 (60-86)	56 (50-70.0)	<0.001**
TG (mg/dL)	96 (64-138)	112 (88-132)	0.905	87 (58-112)	127 (89-160)	<0.001**
LDL-C (mg/dL)	122 (98-144)	114 (94-134)	0.317	122 (99-146)	121 (96-138)	0.066
HbA1c (%)	5.7 (5.4-6.0)	6.0 (5.7-6.1)	0.007**	5.8 (5.5-6.1)	5.8 (5.6-6.1)	0.327
FPG (mg/dL)	102 (95-110)	100¥4 (100-110)	0.229	101 (95-109)	105 (99-110)	0.003**
Insulin (μU/mL)	5.7 (4.1-8.2)	7.5 (4.9-9.9)	0.531	5.5 (4.1-7.3)	7.4 (4.7-9.2)	0.162
HOMA-β	53.2 (35.4-77.3)	63.5 (42.0-86.4)	0.194	54.6 (36.4-72.0)	58.8 (40.5-81.4)	0.054
HOMA-IR	1.5 (1-2.1)	2.0 (1.2-2.5)	0.692	1.4 (1.0-1.9)	1.9 (1.2-2.4)	0.263
TyG index	8.50 (8.04-8.93)	8.65 (8.43-8.93)	0.057	8.35 (7.94-8.71)	8.77 (8.43-9.05)	<0.001**

CRP (mg/dL)	0.05 (0.05-0.13)	0.07 (0.05-0.13)	0.745	0.05 (0.05-0.07)	0.06 (0.05-0.16)	**0.004****
eGFR (mL/min/1.73m^2)	75 (64-84)	73 (64-82)	0.456	77 (68-83)	73 (60-82)	**0.006****

*: $p < 0.05$; **: $p < 0.01$. red indicates higher values for males compared to females.

Supplemental Table S2. Correlation of fasting insulin, FPG, HbA1c, HOMA-b, and HOMA-IR with clinical and laboratory parameters in 207 participants.

		Insulin (μU/mL)			FPG (mg/dL)			HbA1c (%)			HOMA-β			HOMA-IR		
		Total	Male	Female	Total	Male	Female	Total	Male	Female	Total	Male	Female	Total	Male	Female
		n=207	n=100	n=107	n=207	n=100	n=107	n=207	n=100	n=107	n=207	n=100	n=107	n=207	n=100	n=107
Age (years)	*r*	-0.053	-0.148	0.020	0.196	-0.007	0.329	0.313	0.203	0.405	-.162*	-0.178	-0.145	-0.007	-0.129	0.087
	p	0.447	0.142	0.839	0.005**	0.944	<0.001**	<0.001**	0.043*	<0.001**	0.019	0.076	0.136	0.922	0.202	0.371
BMI (kg/m^2)	*r*	0.365	0.475	0.276	0.402	0.236	0.425	0.24	0.221	0.253	0.189	0.385	0.076	0.411	0.487	0.331
	p	<0.001**	<0.001**	0.004	<0.001**	0.018*	<0.001**	<0.001**	0.027*	0.009**	0.006*	<0.001**	0.434	<0.001**	<0.001**	<0.001**
Waist circumference (cm)	*r*	0.378	0.484	0.289	0.383	0.141	0.436	0.272	0.198	0.34	0.208	0.436	0.074	0.417	0.481	0.343
	p	<0.001**	<0.001**	0.003**	<0.001**	0.161	<0.001**	<0.001**	0.048*	<0.001**	0.003**	<0.001**	0.450	<0.001**	<0.001**	<0.001**
TC (mg/dL)	*r*	-0.147	-0.186	-0.086	0.133	0.194	0.204	0.113	-0.057	0.318	-0.2	-0.251	-0.167	-0.108	-0.148	-0.032
	p	0.035*	0.064	0.378	0.056	0.053	0.035*	0.106	0.570	<0.001**	0.004**	0.012*	0.086	0.122	0.141	0.743
HDL-C (mg/dL)	*r*	-0.329	-0.449	-0.255	-0.294	-0.121	-0.303	-0.105	-0.093	-0.069	-0.185	-0.399	-0.076	-0.344	-0.447	-0.277
	p	<0.001**	<0.001**	0.008**	<0.001**	0.231	0.002**	0.132	0.358	0.479	0.008**	<0.001**	0.434	<0.001**	<0.001**	0.004**
TG (mg/dL)	*r*	0.21	0.236	0.170	0.338	0.115	0.459	0.19	0.066	0.308	0.048	0.189	-0.070	0.245	0.241	0.231
	p	0.002**	0.018*	0.081	<0.001**	0.254	<0.001**	0.006**	0.516	0.001**	0.492	0.059	0.473	<0.001**	0.016*	0.017*
LDL-C (mg/dL)	*r*	-0.013	-0.097	0.068	0.259	0.284	0.31	0.145	0.020	0.268	-0.123	-0.173	-0.089	0.034	-0.050	0.127
	p	0.848	0.336	0.486	<0.001**	0.004**	0.001**	0.037*	0.843	0.005**	0.078	0.085	0.362	0.624	0.625	0.192
Ferritin (ng/mL)	*r*	0.168	0.179	0.147	0.054	-0.049	-0.071	-0.081	-0.200	-0.011	0.127	0.172	0.158	0.172	0.167	0.127
	p	0.016*	0.075	0.131	0.442	0.631	0.469	0.246	0.046*	0.909	0.069	0.086	0.104	0.013*	0.097	0.193
RBC ($\times 10^4$/μL)	*r*	0.223	0.314	0.106	0.218	0.183	0.022	0.190	0.226	0.142	0.136	0.260	0.084	0.237	0.321	0.100

	p	0.001**	0.001**	0.275	0.002**	0.068	0.819	0.006**	0.024*	0.145	0.051	0.009**	0.389	<0.001**	0.001**	0.304
WBC (/μL)	*r*	0.211	0.168	0.280	0.202	0.135	0.163	0.118	0.158	0.060	0.115	0.100	0.179	0.237	0.173	0.306
	p	0.002**	0.095	0.003**	0.004**	0.181	0.094	0.090	0.116	0.538	0.099	0.321	0.066	<0.001**	0.085	0.001**
Plt (×10⁴/μL)	*r*	0.064	0.110	0.050	0.012	0.073	-0.002	0.070	0.149	-0.001	0.062	0.061	0.065	0.060	0.115	0.038
	p	0.359	0.276	0.610	0.869	0.472	0.980	0.317	0.139	0.992	0.375	0.549	0.503	0.391	0.254	0.697
CRP (mg/dL)	*r*	0.222	0.216	0.238	0.164	0.023	0.182	0.179	0.171	0.173	0.112	0.168	0.107	0.239	0.205	0.266
	p	0.002**	0.033*	0.015*	0.019*	0.825	0.064	0.011*	0.092	0.080	0.111	0.099	0.281	<0.001**	0.043*	0.006**
eGFR (mL/min/1.73m²)	*r*	-0.029	0.003	-0.017	-0.097	-0.028	-0.029	-0.173	-0.232	-0.067	0.001	-0.018	0.001	-0.047	-0.001	-0.029
	p	0.679	0.974	0.862	0.162	0.782	0.763	0.012*	0.020*	0.494	0.987	0.858	0.989	0.502	0.988	0.766

*: $p < 0.05$; **: $p < 0.01$. Blue indicates lower values for males compared to females, red indicates higher values for males compared to females.
